# Dynamics robustness of cascading systems

**DOI:** 10.1371/journal.pcbi.1005434

**Published:** 2017-03-13

**Authors:** Jonathan T. Young, Tetsuhiro S. Hatakeyama, Kunihiko Kaneko

**Affiliations:** Research Center for Complex Systems Biology, The University of Tokyo, Tokyo, Japan; University of Illinois at Urbana-Champaign, UNITED STATES

## Abstract

A most important property of biochemical systems is robustness. Static robustness, e.g., homeostasis, is the insensitivity of a state against perturbations, whereas dynamics robustness, e.g., homeorhesis, is the insensitivity of a dynamic process. In contrast to the extensively studied static robustness, dynamics robustness, i.e., how a system creates an invariant temporal profile against perturbations, is little explored despite transient dynamics being crucial for cellular fates and are reported to be robust experimentally. For example, the duration of a stimulus elicits different phenotypic responses, and signaling networks process and encode temporal information. Hence, robustness in time courses will be necessary for functional biochemical networks. Based on dynamical systems theory, we uncovered a general mechanism to achieve dynamics robustness. Using a three-stage linear signaling cascade as an example, we found that the temporal profiles and response duration post-stimulus is robust to perturbations against certain parameters. Then analyzing the linearized model, we elucidated the criteria of when signaling cascades will display dynamics robustness. We found that changes in the upstream modules are masked in the cascade, and that the response duration is mainly controlled by the rate-limiting module and organization of the cascade’s kinetics. Specifically, we found two necessary conditions for dynamics robustness in signaling cascades: 1) Constraint on the rate-limiting process: The phosphatase activity in the perturbed module is not the slowest. 2) Constraints on the initial conditions: The kinase activity needs to be fast enough such that each module is saturated even with fast phosphatase activity and upstream changes are attenuated. We discussed the relevance of such robustness to several biological examples and the validity of the above conditions therein. Given the applicability of dynamics robustness to a variety of systems, it will provide a general basis for how biological systems function dynamically.

## Author summary

Cells use signaling pathways to transmit information received on its membrane to DNA, and many important cellular processes are tied to signaling networks. Past experiments have shown that cells’ internal signaling networks are sophisticated enough to process and encode temporal information such as the length of time a ligand is bound to a receptor. However, little research has been done to verify whether information encoded onto temporal profiles can be made robust. We examined mathematical models of linear signaling networks and found that the relaxation of the response to a transient stimuli can be made robust to certain parameter fluctuations. Robustness is a key concept in biological systems—it would be disastrous if a cell could not operate if there was a slight change in its environment or physiology. Our research shows that such dynamics robustness is a property of linear signaling cascades, and we outline the design principles needed to generate such robustness. We discovered that two conditions regarding the speed of the internal chemical reactions and concentration levels are needed to generate dynamics robustness.

## Introduction

Robustness is one of the most important concepts in biological systems. In general, it is the ability of an organism to maintain a state or behavior against external or internal perturbations, and many frameworks of robustness have emerged [1–7]. Homeostasis, for example, is the ability of an organism or a cell to maintain a certain state, such as its body temperature or calcium content, against external environmental changes. In fact, numerous mechanisms have been uncovered that are adopted to regulate its internal environment against external perturbations. In developmental biology, differentiated cellular states are known to be robust to disturbances, as was pioneered in the study by Waddington, who described the cell differentiation process as a ball rolling down an epigenetic landscape to settle into a stable valley [8]. This is a metaphorical representation of robustness often used, while in terms of dynamical systems theory, one mathematical formulation for static robustness can be described as an orbit being pulled into a stable attractor. The robustness discussed therein is concerned about the stationary state, and thus is regarded as *static robustness*.

In biology, however, both the static cellular states and dynamic processes are important to make certain responses against external changes robust and to ensure proper development. Waddington coined the term homeorhesis for such dynamics robustness for a transient time course [9]. Indeed, in the developmental process, temporal ordering of cell differentiations and their timing are robust. Besides the developmental process, cellular responses against external stimuli are often robust to perturbations since these time courses are often relevant to cellular function. Despite the importance, such *dynamics robustness*, i.e., robustness in the temporal course, is little understood as compared with extensive studies on static robustness. Here we study dynamics robustness, the insensitivity of transients to initial conditions or parameters. We adopt the term *dynamics robustness* as opposed to dynamic or dynamical robustness since those terms have been defined elsewhere in a different context. For example in [10], dynamic robustness refers to the insensitivity of a steady-state against changes in protein concentrations to distinguish from the robustness of a steady-state against gene deletions. We stress that our focus is on the robustness of the dynamics themselves against parameter perturbations.

As a specific example for such robustness, we focus on signaling pathways of covalent modification cycles. Indeed, robustness therein has been extensively investigated as given by a recent review by Blüthgen and Legewie [11]. Although their review is focused on static robustness in signal transduction pathways, they also note that ideas of robustness with regards to generating an invariant temporal profile (dynamics robustness) has to be developed. In fact, there are several experiments suggesting robustness in the transient properties of certain biochemical networks: Different transient profiles of input stimuli can elicit different phenotypic responses. For example, it was shown that the duration of activation could lead to two different responses in PC12 cells; transient activation leads to proliferation, and sustained activation leads to differentiation. In a similar manner, the duration that a MAPK cascade is stimulated can lead to different responses in yeast. Moreover, temporal profiles of the p53 pathway, which is inactivated in almost all human cancer cells, are also reported to be drastically altered by the types of stresses administered to the cells and cause different responses depending on the dynamic profiles. Indeed, there are numerous other examples about the importance of transient dynamics in SSH signaling [12], NF-*κ*B [13], and metabolism [14]. All of these experimental reports suggest the need for studies on dynamics robustness. Beyond such experimental results, research in the last decade has shown that signaling cascades can theoretically encode information into their dynamic profiles and process such information as well. For these dynamical processes to function, the time courses need to achieve a certain level of robustness.

We investigated a class of signaling cascade systems and examined *duration robustness* as a quantitative manifestation of dynamics robustness, wherein the duration of a response upon inputs is robust against perturbations. In a general class of cascading systems, we showed that duration robustness is an intrinsic property: Downstream modules are shielded from perturbations in the enzymatic activity in the upstream layers. Here, the organization of the fast and slow kinetics resulting in a rate-limiting module is primarily responsible for such robustness. In a linear signaling system, by having fast kinase activity, the output time courses were shown to be robust to perturbations in the phosphatase activity. We uncovered two necessary conditions for dynamics robustness and demonstrated that it can be observed in general linear signaling systems via protein modifications. Furthermore, we verified that dynamics robustness is a property of the well-known model of a MAPK network described by Huang and Ferrell [15].

## Results

**Fig 1:**
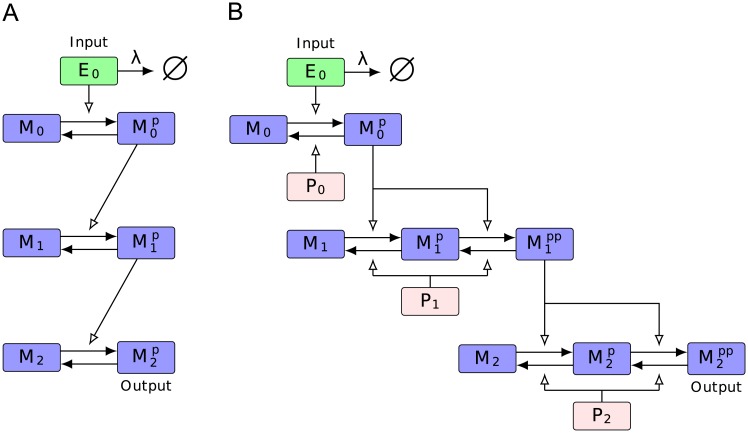
Diagrams of the Heinrich and Huang-Ferrell Model. A linear signaling cascade is a biochemical network where the product of one reaction acts as an enzyme for a reaction downstream. (A) The Heinrich model captures the basic essence of such an architecture. For time *t* < 0, the receptor, *E*_0_ receives a stimulus with strength $E_{0}^{init}$. *E*_0_ then converts *M*_0_ to $M_{0}^{p}$. $M_{0}^{p}$ then converts *M*_1_ to $M_{1}^{p}$, and $M_{1}^{p}$ converts *M*_2_ to $M_{2}^{p}$. The concentration of $M_{2}^{p}$ is considered the output response. After the system reaches a steady-state, at time *t* = 0, the stimulus is immediately removed, and the system then settles into a deactivated state. (B) The Huang-Ferrell is a more complicated model that explicitly includes the phosphatase at each layer and assumes mass-action kinetics. The second and third layer also assume double-phosphorylation events are needed for activation.

Our results are organized as follows: We study a simple model of a basic linear signaling cascade and see how perturbing the parameters in the model affect the relaxation time courses. We first focus on perturbing the phosphatase parameters because it has been reported that phosphatase activity controls the duration more than the kinase activity [16, 17]. We then show how perturbing the kinase activities affect the results. Next, we analyze the linearized and normalized model of the aforementioned basic linear cascade to determine what underlying features of the cascade architecture causes dynamics robustness. From this analysis, we derive the conditions under which dynamics robustness is expected. Finally, we examine a more complicated mass-action model of a MAPK cascade to verify whether the results observed in the simple model are indeed features of a more biologically inspired model. Diagrams of the models we examined are given in Fig 1.

We measure dynamics robustness using the Euclidean distance between a temporal profile and the profile after a parameter perturbation. We will describe this measure in more detail later. As a simpler, more analytically tractable measure for robustness, we also looked at whether the duration of the response is robust. We note that dynamics robustness implies duration robustness, but the converse is not necessarily true. As in [16], we define the duration of the response to be its half-life, which we label as *ϑ*. We consider the duration to be robust against a parameter perturbation if the logarithmic gain (see Methods Section) is small. A linear logarithmic gain (as expected with a relaxation of the form exp(−*βt*)) would have a magnitude of 1, and so we define the threshold of robustness to be 0.3. This choice is somewhat arbitrary, but our results do not change if a reasonable threshold is selected.

### Dynamics robustness in the Heinrich model

We first examined the Heinrich model of a general, linear signaling cascade (a detailed description can be found in the Methods Section). The basic idea is that a stimulus, the concentration of *E*_0_, activates a kinase, i.e., converts *M*_0_ to $M_{0}^{p}$, which goes on to activate a kinase downstream. This process occurs in three steps, and the concentration of the final activated kinase, $M_{2}^{p}$, is considered the output response.

**Fig 2:**
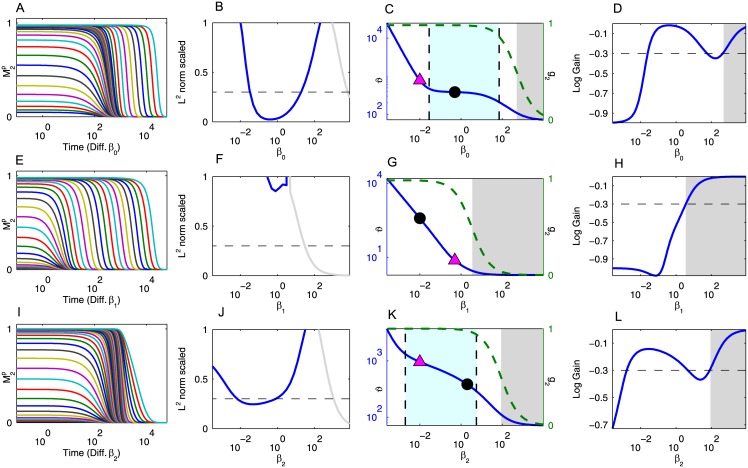
Results for the Heinrich Model. (A, E, I) The temporal profiles of the response relaxation for different values of *β*_0_, *β*_1_, and *β*_2_. In (A), we set *β*_1_ and *β*_2_ equal to their base values from Table B in S1 Appendix. We then integrated the Heinrich model for the *β*_0_ values taken from the set $\left\{\beta_{0}^{0},\beta_{0}^{1},\ldots ,\beta_{0}^{37},\beta_{0}^{38}\right\}=\left\{10^{4.0},10^{3.8},\ldots ,10^{-3.4},10^{-3.6}\right\}$. (B, F, J) The consecutive similarity in the temporal profiles for *β*_0_, *β*_1_, and *β*_2_. We consider the system to display dynamics robustness against a parameter if this measure is less than 0.3. The gray portion of the lines in (B, F, J) indicate that the system is in a deactivated state (i.e., *g*_2_ < 0.5) for those values of *β*_*i*_. (C, G, K) The half-life of the response as a function of *β*_0_, *β*_1_, and *β*_2_. The magenta triangle indicates when the *β*_*i*_ value becomes the minimum *β* value. The black dot represents the base *β*_*i*_ value from Table B in S1 Appendix. The grayed out region indicates that the system is in a deactivated state for those values of *β*_*i*_. The region between the dashed vertical lines indicate that the magnitude logarithmic gain of the duration against *β*_*i*_ is less than 0.3. The dashed green line is *g*_2_ as a function of *β*_*i*_. (D, H, L) The logarithmic gain of the duration against *β*_0_, *β*_1_, and *β*_2_.

When time *t* < 0, a constant input $E_{0}^{init}$ is applied to the system until $M_{i}^{p}$ at each layer reaches the steady-state concentration, which we define as $\tilde{M_{i}}$. At time *t* = 0, *E*_0_ is set equal to zero and the system begins to relax into a deactivated state. We individually perturbed the total phosphatase activity at each layer and computed the new temporal profile to see if it remains robust. The parameters were chosen to reflect the same organization as the biologically relevant MAPK cascade parameters reported in [15] (see Supporting Information); the kinase activities are relatively fast, and the phosphatase rate constants are organized relatively as fast-slow-fast in the three stage setup. The specific *β* values from this parameter set correspond to the black circles in Fig 2(C), 2(G) and 2(K). For clarity, the Heinrich model parameter definitions are given in Table 1.

**Table 1:** Parameters in the Heinrich model.

$E_{0}^{init}$	Initial input strength at time *t* < 0
*M*_*i*_	Unphosphorylated substrate in the *i*^*th*^ module
$M_{i}^{p}$	Phosphorylated substrate in the *i*^*th*^ module
$M_{i}^{tot}$	Total substrate in the *i*^*th*^ module ($M_{i}+M_{i}^{p}$)
${\tilde{M}}_{i}$	Steady-state value of $M_{i}^{p}$ under $E_{0}=E_{0}^{init}$
*α*_*i*_	Effective kinase activity in the *i*^*th*^ module
*β*_*i*_	Effective phosphatase activity in the *i*^*th*^ module
*g*_*i*_	Initial phosphorylation level of *i*^*th*^ module (${\tilde{M}}_{i}/M_{i}^{tot}$)
*ϑ*	Half-life of response

In this paper, we focus our discussion on the relaxation process of strongly activated cascades because the dynamics of a weakly activated signaling cascade are fundamentally different, and do not involve a significant relaxation time course. To clearly describe the criterion of activation, we introduce the initial steady-state value $g_{i}=\tilde{M_{i}}/M_{i}^{tot}$, which is the ratio of the phosphorylated substrate to the total substrate in *i*^*th*^ module for a given $E_{0}^{init}$. As we are interested in the response dynamics of the cascade, the initial activation *g*_2_ should be sufficiently high. Henceforth, we use the criterion that the cascade is activated if *g*_2_ > 0.5 (although the value 0.5 itself is not essential).

The results for the Heinrich model are plotted in Fig 2. There is an interesting parameter region where the temporal profiles are close together despite *β*_0_ decreasing from 10^2^ to 10^−2^ (Fig 2(A)). There is a similar parameter region for *β*_2_ (Fig 2(I)). In a certain range of *β*_0_ and *β*_2_, the temporal profiles do not change based on the phosphatase activity. However, there is no such parameter region in which the temporal profiles are not changed when *β*_1_ is perturbed (Fig 2(E)). To measure how close the time-course profiles are when changing *β*_0_, we used the *L*^2^ norm between consecutive temporal profiles from Fig 2(A). In other words, we computed

$$\frac{1}{{\text{log}}_{10}\left(\beta_{0}^{i}\right)-{\text{log}}_{10}\left(\beta_{0}^{i+1}\right)}{\left\|M_{2}^{p}\left(\tau ;\beta_{0}^{i}\right)-M_{2}^{p}\left(\tau ;\beta_{0}^{i+1}\right)\right\|}_{2}=\frac{1}{{\text{log}}_{10}\left(\beta_{0}^{i}\right)-{\text{log}}_{10}\left(\beta_{0}^{i+1}\right)}\sqrt{\overset{b}{\underset{a}{\int }}{\left|M_{2}^{p}\left(\tau ;\beta_{0}^{i}\right)-M_{2}^{p}\left(\tau ;\beta_{0}^{i+1}\right)\right|}^{2}d\tau },$$

where *τ* = log_10_(*t*) and the integral is approximated over the interval [−2, 4 + log_10_(6)]. If this measure is less than 0.3, then we consider the temporal profiles to be robust against perturbations. We stress again that 0.3 was chosen arbitrarily, but any reasonable threshold will work. We performed a similar analysis for *β*_1_ and *β*_2_ in Fig 2(F) and 2(J). The temporal profiles of the output, $M_{2}^{p}$, show dynamics robustness against changes in the phosphatase activity in the first and third layers, i.e., the time course profiles are robust to perturbations in *β*_0_ and *β*_2_. (Here we used the scaled *L*^2^ norm of perturbed temporal profiles as measures of robustness, but other measures may also be feasible. For example, the Kullback-Leibler [18] or other information oriented measures may be adopted.)

We plotted the half-life duration, *ϑ*, as a function of *β*_*i*_ on a log-log scale in Fig 2(C), 2(G) and 2(K). We color the inactivated region in gray in Fig 2(C), 2(G) and 2(K) and focus on the dynamics in the region of strong activation. The regions between the dashed vertical lines in Fig 2(C) and 2(K)) represent where the magnitude of the logarithmic gain is less than 0.3, which is distinctly smaller than 1. These flatter slopes indicate that the duration is robust against changes in *β*_0_ and *β*_2_. For clarity, we plotted the logarithmic gain in Fig 2(D), 2(H) and 2(L).

In Fig 2(C) and 2(K), the black circle, which represents the *β*_*i*_ value from Table B in S1 Appendix and the corresponding *ϑ* value, is in the region of duration robustness, which means that with this parameter set reflecting actual kinetics in signaling cascades, the duration is robust to perturbations in the phosphatase activity in the first and last layer of the cascade (*β*_0_ and *β*_2_ respectively). However, the second module is sensitive to perturbations in the phosphatase activity.

In all three cases, there are common features in the plots of the duration. As mentioned earlier, if the phosphatase activity in any layer is too high, then the system is in an inactivated state, which is colored in gray in Fig 2(C), 2(G) and 2(K). On the other hand, if the phosphatase activity in the *i*^*th*^ layer is too low, then the logarithmic gain of the duration against *β*_*i*_ is roughly −1, i.e., the duration of the response is strongly dependent on the rate-limiting module in the cascade. In Fig 2(C), 2(G) and 2(K) we plotted a magenta triangle at the value where *β*_*i*_ becomes less than all other *β* values, and the logarithmic gain indeed becomes −1 near this point. However, the upper bound for the phosphatase concentration that exhibits duration robustness cannot be described by the rate-limiting effect only.

### Effects of the kinase activity on duration robustness

**Fig 3:**
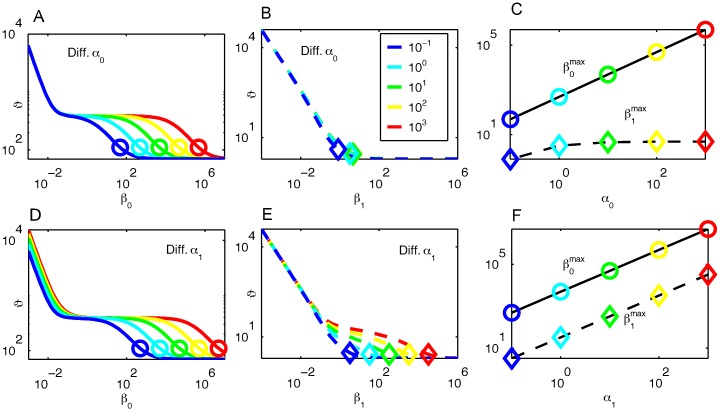
Effects of the Kinase Activity on Duration Robustness in the Heinrich Model. (A, B) Duration, *ϑ*, as a function of *β*_0_ and *β*_1_ with varied *α*_0_. Different lines indicate *ϑ* for different *α*_0_ values as given by the inset box in (B). Circles and diamonds represent $\beta_{0}^{max}$ and $\beta_{1}^{max}$, respectively. (C) $\beta_{i}^{max}$ as a function of *α*_0_. A solid line and a dashed line are $\beta_{0}^{max}$ and $\beta_{1}^{max}$, respectively. The circles and the diamonds correspond to these symbols in (A) and (B). (D, E) Duration, *ϑ*, as a function of *β*_0_ and *β*_1_ with varied *α*_1_. (F) $\beta_{i}^{max}$ as a function of *α*_1_. Same colors, lines and symbols are adopted as (A, B, C).

Although linear signaling cascades can show duration robustness against perturbations in the phosphatase concentrations, it is still unclear what effects the kinase activity has. Therefore, we computed the duration versus phosphatase activity (*ϑ* vs. *β*_*i*_) for different values of *α*_*i*_ (Fig 3).

Increasing *α*_0_ expands the interval of duration robustness for *β*_0_, since the upper limit is increased while the lower limit remains fixed (Fig 3A). This increase of *α*_0_, however, does not expand the duration for varied *β*_1_ (Fig 3B). On the other hand, increasing *α*_1_ expands the interval of duration robustness both for *β*_0_ and for *β*_1_: the slope of *ϑ* against *β*_1_ is flatter, resulting in the appearance of the region for duration robustness for *β*_1_.

Here, the upper limit of duration robustness is roughly given by the largest value of *β*_*i*_, which we call $\beta_{i}^{max}$, at which the system is activated. The $\beta_{i}^{max}$ values are marked in Fig 3(A), 3(B), 3(D) and 3(E). By using the criterion of *g*_2_, $\beta_{i}^{max}$ is given by the maximal value of *β*_*i*_ that satisfies *g*_2_(*β*_*i*_) > 0.5. $\beta_{i}^{max}$ is then used as an indicator for the upper limit of the interval of duration robustness. To derive an expression for $\beta_{i}^{max}$, we see that *g*_*i*_, the initial steady-state phosphorylation level at each stage, can be written as a sequence of iterations:

$$\begin{array}{cc} g_{0} & =\frac{1}{1+\frac{\beta_{0}}{\alpha_{0}}},\\ g_{1} & =\frac{1}{1+\frac{\beta_{1}}{\alpha_{1}g_{0}}}=\frac{1}{1+\frac{\beta_{1}}{\alpha_{1}}\left(1+\frac{\beta_{0}}{\alpha_{0}}\right)},\\ g_{2} & =\frac{1}{1+\frac{\beta_{2}}{\alpha_{2}g_{1}}}=\frac{1}{1+\frac{\beta_{2}}{\alpha_{2}}\left(1+\frac{\beta_{1}}{\alpha_{1}}\left(1+\frac{\beta_{0}}{\alpha_{0}}\right)\right)}. \end{array}$$ (1)

If changes in the kinase activity cause changes in $\beta_{i}^{max}$, then the region of duration robustness will change as seen in Fig 3. The iterative nature of Eq 1 demonstrates how upstream parameter changes are shielded. It clearly shows that increasing *α*_*k*_ will proportionally increase $\beta_{i}^{max}$ *only* for *i* ≤ *k*. For *i* > *k*, increasing *α*_*k*_ has a negligible effect on $\beta_{i}^{max}$, and hence, has a negligible effect on the interval of duration robustness for *β*_*i*_. This is somewhat counterintuitive because one usually considers alterations propagating downstream in a linear signaling cascade, whereas alterations in the kinase activity affects the range of duration robustness only in upstream modules. This is because the constraint for initial conditions back-propagates. In general, having fast kinase activity in the downstream modules is ideal if one wishes to generate a region of duration robustness against the upstream phosphatase activities.

### Duration robustness in a linearized model

**Fig 4:**
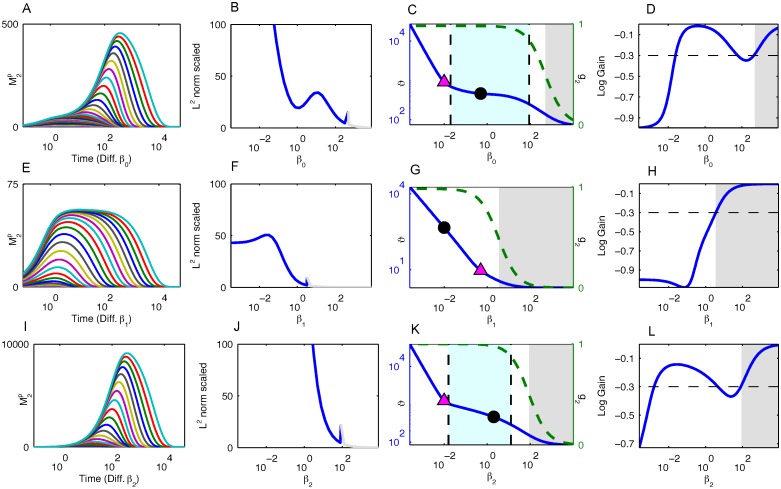
Results for the Linearized Heinrich Model. (A, E, I) The temporal profiles of the response relaxation for different values of *β*_0_, *β*_1_, and *β*_2_. The same parameters for Fig 2 are used here. (B, F, J) The consecutive similarity in the temporal profiles for *β*_0_, *β*_1_, and *β*_2_. At no point does the system display dynamics robustness. The gray portion of the lines in (B, F, J) indicate that the system is in a deactivated state (i.e., *g*_2_ < 0.5) for those values of *β*_*i*_. (C, G, K) The half-life of the response as a function of *β*_0_, *β*_1_, and *β*_2_. The magenta triangle indicates when the *β*_*i*_ value becomes the minimum *β* value. The black dot represents the base *β*_*i*_ value from Table B in S1 Appendix. The grayed out region indicates that the system is in a deactivated state for those values of *β*_*i*_. The region between the dashed vertical lines indicate that the magnitude logarithmic gain of the duration against *β*_*i*_ is less than 0.3. The dashed green line is *g*_2_ as a function of *β*_*i*_. (D, H, L) The logarithmic gain of the duration against *β*_0_, *β*_1_, and *β*_2_.

To better understand how duration robustness is generated and the criteria needed, we analyzed the linearization of the Heinrich model about the origin, the only equilibrium point once the stimulus is removed. Duration robustness is also a property of the linear model as can be seen in Fig 4. The global linearization of the Heinrich model is a significant departure, and the time-course profiles in Fig 4(A), 4(E) and 4(I) for the linear case are drastically different from the ones for the nonlinear Heinrich model in Fig 2(A), 2(E) and 2(I). In particular, the conserved quantities in the nonlinear model are no longer conserved in the linear model. However, the plots of the duration against the phosphatase activity in Fig 4 are remarkably similar to those in Fig 2. This strongly suggests that the nonlinear kinetics are not important for duration robustness, although we will show that the nonlinearity of *g*_2_ as a function of *β*_*i*_ does play a crucial role.

If *β*_0_ ≠ *β*_1_ ≠ *β*_2_, the normalized solution ($m_{i}^{p}=M_{i}^{p}/{\tilde{M}}_{i}$) is just a linear combination of exponentials:

$$m_{2}^{p}\left(t\right)=c_{0}\left(\beta_{i},\alpha_{i}\right)e^{-\beta_{0}t}+c_{1}\left(\beta_{i},\alpha_{i}\right)e^{-\beta_{1}t}+c_{2}\left(\beta_{i},\alpha_{i}\right)e^{-\beta_{2}t}.$$

The duration (the time *ϑ* such that $m_{2}^{p}\left(\vartheta \right)=0.5$) can be approximated by:

$$\vartheta \approx \left\{\begin{array}{cc} \frac{1}{\beta_{0}}\log\left(2c_{0}\left(\beta_{i},\alpha_{i}\right)\right) & \text{if}\;\beta_{0}<\beta_{1},\beta_{2},\\ \frac{1}{\beta_{1}}\log\left(2c_{1}\left(\beta_{i},\alpha_{i}\right)\right) & \text{if}\;\beta_{1}<\beta_{0},\beta_{2},\\ \frac{1}{\beta_{2}}\log\left(2c_{2}\left(\beta_{i},\alpha_{i}\right)\right) & \text{if}\;\beta_{2}<\beta_{0},\beta_{1}. \end{array}\right.$$ (2)

The pertinent question is how *ϑ* is made robust to changes in *β*_*i*_. If *β*_*i*_ is the minimum *β* value, then the duration according to Eq 2 is roughly inversely proportional to *β*_*i*_, which means that the logarithmic gain is going to be around −1. In fact, in the limit as *β*_*i*_ goes to 0, the logarithmic gain converges to −1. In this case, accordingly, there is no duration robustness. Hence, to have duration robustness against *β*_*i*_, the first constraint is

$$\min\left\{\beta_{j}\right\}<\beta_{i},$$ (3)

which we refer to as the *constraint on the rate-limiting process*.

The lower limit of the *β*_*i*_ interval for duration robustness is determined by this rate-limiting condition; however, this condition is not sufficient to determine the upper limit of the interval of *β*_*i*_. As already discussed, the initial phosphorylation level *g*_2_ at the output layer has to be sufficiently activated, and as shown in Fig 4, the upper limit is strongly related to this initial phosphorylation level *g*_2_. Indeed, we can use Eq 2 to understand this behavior analytically. Suppose that *β*_*k*_ is the minimum *β* value and that *i* ≠ *k*. Then the logarithmic gain is given by:

$$\frac{\partial \log(\vartheta )}{\partial \log(\beta_{i})}=\frac{1}{\log\left(2\right)+\log\left(c_{k}\right)}\frac{\partial \log(c_{k})}{\partial \log(\beta_{i})}.$$ (4)

Therefore, if the logarithmic gain of *c*_*k*_ with respect to *β*_*i*_ is small, then the cascade will display duration robustness. As shown in the Supporting Information, $\frac{\partial \log(c_{k})}{\partial \log(\beta_{i})}$ is strongly dependent on $-\frac{\partial \log(g_{2})}{\partial \log(\beta_{i})}$.

If *g*_2_ has a sigmoidal nature as seen by the dashed green lines in Fig 4(C), 4(G) and 4(K), then it has two regions where it is relatively constant with respect to *β*_*i*_ and a transition state between the two relatively constant regions. If this transition occurs before the module becomes rate limiting, then duration robustness will exist because *g*_2_ will have a weak dependence on *β*_*i*_. As mentioned previously in relationship with Eq 1, changes in upstream kinase activity have a negligible effect on *g*_2_, i.e., upstream parameter changes are shielded. Hence, to increase the transition point and expand the region of duration robustness in the *i*^*th*^ module, it is necessary that there exists some *j* ≥ *i* such that $\beta_{j}\ll \alpha_{j}$. In other words, it is necessary that in a module downstream, the kinase activity relative to the phosphatase activity needs to be very fast. We refer to this constraint regarding *g*_2_ as the *constraint on initial conditions*.

The arguments based on Eqs 1 and 2 can be extended to an N-stage cascade, and the conditions needed to generate duration robustness in the *i*^*th*^ module can be summarized as

$$\begin{array}{cc} \min\{\beta_{j}\} & <\beta_{i},\\ \frac{\beta_{N}}{\alpha_{N}}\left(1+\frac{\beta_{N-1}}{\alpha_{N-1}}\left(\cdots \left(1+\frac{\beta_{0}}{\alpha_{0}}\right)\right)\right) & <1,\\ \exists k\geq i\;\text{such}\;\text{that}\;\beta_{k} & \ll \alpha_{k}, \end{array}$$ (5)

where the first condition represents the constraint on the rate-limiting process, and the latter two conditions give the constraint on the initial conditions.

The arguments made for the linearization can also be extended to general linear signaling cascades. The rate-limiting condition can easily be understood using slow manifold theory. The eigenmodes of a linear signaling cascade are proportional to the phosphatase activity. Likewise, the phosphorylation levels at each stage do display a switch-like nature. Because the kinase activity controls the phosphorylation levels, both constraints, i.e., the rate-limiting condition and the constraint on the initial conditions, will also be necessary in any model of a linear signaling cascade.

While both the original and linearized Heinrich models display duration robustness, the original Heinrich model displays a stronger type of dynamics robustness in the sense that the time-course profiles themselves are robust to changes in *β*_*i*_ under certain conditions (see Fig 2(A), 2(E) and 2(I)). This is mainly because in the linear model, the response is unsaturated and can vary, whereas the response in the nonlinear model is saturated, bounded, and decreasing for all relevant parameter regimes.

### Dynamics robustness in the Huang Ferrell model

To verify the general results on a more biologically inspired system, we next examined the Huang Ferrell (HF) model of a linear signaling cascade (for a detailed description, see Supporting Information). This model is a complete mass action description of a MAPK signaling pathway, which is a linear cascade with three layers. The middle and last layers represent double phosphorylation events, which lead to ultrasensitivity. The HF model also explicitly assumes that a phosphatase at each layer removes the active phosphate groups, and thus, the phosphatase activity is directly proportional to the total phosphatase concentration, $P_{i}^{tot}$, in each layer.

**Fig 5:**
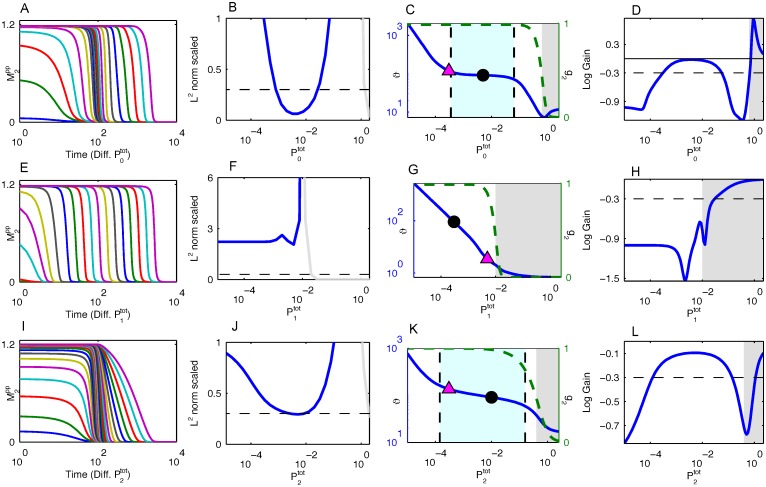
Results for the HF Model. (A, E, I) The temporal profiles of the response relaxation for different values of $P_{0}^{tot}$, $P_{1}^{tot}$, and $P_{2}^{tot}$. In Fig 5(A), we set $P_{1}^{tot}$ and $P_{2}^{tot}$ equal to their base values from Table A in S1 Appendix. We then integrated the HF model for each of the $P_{0}^{tot}$ values taken from the set $\left\{P_{0}^{tot,0},P_{0}^{tot,1},\ldots ,P_{0}^{tot,26},P_{0}^{tot,27}\right\}=\left\{10^{0.4},10^{0.2},\ldots ,10^{-4.8},10^{-5.0}\right\}$. (B, F, J) The consecutive similarity in the temporal profiles for $P_{0}^{tot}$, $P_{1}^{tot}$, and $P_{2}^{tot}$. In Fig 5(B), we computed the *L*^2^ norm in the difference between consecutive temporal profiles. The gray portion of the lines in (B, F, J) indicate that the system is in a deactivated state (i.e., *g*_2_ < 0.5) for those values of $P_{i}^{tot}$. (C, G, K) The half-life of the response as a function of $P_{0}^{tot}$, $P_{1}^{tot}$, and $P_{2}^{tot}$. The magenta triangle indicates when the $P_{i}^{tot}$ value becomes the minimum *P*^*tot*^ value. The black dot represents the base $P_{i}^{tot}$ value from Table A in S1 Appendix. The grayed out region indicates that the system is in a deactivated state for those values of $P_{i}^{tot}$. The region between the dashed vertical lines indicate that the logarithmic gain magnitude of the duration against $P_{i}^{tot}$ is less than 0.3. The dashed green line is *g*_2_ as a function of *β*_*i*_. (D, H, L) The logarithmic gain of the duration against $P_{0}^{tot}$, $P_{1}^{tot}$, and $P_{2}^{tot}$.

The same numerical analysis as for the Heinrich model was performed for the HF model and the results are displayed in Fig 5. The results in Fig 5 demonstrate that duration robustness is also a property of the HF model. There are parameter regimes where the duration of the relaxation is insensitive to perturbations. Like the original Heinrich model, the HF model also displays dynamics robustness in which the time-course profiles themselves are robust to changes in the phosphatase concentrations. By comparing the results in Fig 5 with those in Fig 2, we see that the last layer in the HF model has slightly stronger dynamics robustness than the Heinrich model. This suggests that higher nonlinearities in signaling cascades may enhance dynamics robustness.

## Discussion

In the present paper, we have demonstrated that *dynamics robustness*, i.e., the insensitivity of the time courses against changes in certain parameters, is observed in the relaxation process of signaling cascades. By using a general linear cascading system, the time course of the output layer downstream is shown to be almost insensitive to changes in upstream parameters. As a consequence of dynamics robustness, the duration in which the activated state lasts is also robust to parameter changes, a property we termed *duration robustness*. By analyzing the cascading process, the conditions for duration robustness are given by the constraints on the rate-limiting process and on the initial conditions. Since multiple layers are needed to generate duration robustness, this suggests that this property is a byproduct of how temporal information is processed downstream.

The robustness of flux at steady state has been extensively studied since the pioneering study of Kacser-Burns [19], but a theory for dynamics robustness has not been developed. It will be important to extend the steady-state flux theory to transient dynamics. Here we looked at whether an entire temporal profile can be made robust to parameter perturbations with an analysis similar to [19], which strives to identify which parameters control fluxes in a biochemical network.

### Conditions for duration robustness

We have shown that linear signaling cascades of varying complexity display duration robustness against perturbations in the phosphatase activity in the *i*^*th*^ stage, and that two main conditions are responsible for this phenomenon:

**1) The constraint on the rate-limiting process.** The phosphatase activity in the *i*^*th*^ stage, *β*_*i*_, should not be the minimum *β* value. This unfortunately means that the slowest module in a linear cascade will not display duration robustness. This constraint determines the lower limit for the range of duration robustness, i.e., *β* < *β*_*i*_. If *β*_*i*_ is the minimum *β* value in the cascade, then the duration time is inversely proportional to *β*_*i*_ as described by usual relaxation processes. In general linear signaling cascades, this means that the phosphatase activity in module *i* should not be the slowest. For certain parameter regions, our results are contrary to the idea that upstream phosphatase activity controls the duration of the system more than downstream in a strongly activated cascade [16].
**2) The constraint on the initial conditions.** To achieve duration robustness, the initial phosphorylation level of the output layer also has to be robust. For the Heinrich model, the initial steady-state phosphorylation level, *g*_*i*_, is given as a sequence of iterations as Eq 1, and if the kinase activity in some layer is sufficiently high, *g*_*i*_ will be robust against changes in the upstream phosphatase activity. In other words, changes in upstream layers are shielded by the strong kinase activity. This constraint determines the upper limit for duration robustness.

Intuitively, if the kinase activity is low, the phosphatase activity should be low enough to allow the cascade to be active. How large *β*_*i*_ can be is largely determined by the kinase activity, *α*_*i*_. A stronger kinase activity allows the phosphatase to be at a higher level and the system to remain in an activated state. Although too low kinase activity changes the initial phosphorylation level, too high kinase activity has little effect, due to the saturation of the phosphorylation. This determines the upper limit of *β*_*i*_ for the region of duration robustness.

In general, linear signaling cascades do display such saturation, as a result of conservation of the substrate at each layer, and as for the Heinrich model, the initial steady-state phosphorylation level in *i*^*th*^ layer could be given as

$$g_{i}=\frac{1}{1+f_{i}\left(g_{i-1},\alpha_{i},\beta_{i}\right)},$$

where, *f*_*i*_(*g*_*i*−1_, *α*_*i*_, *β*_*i*_) is a decreasing function of the kinase activity, *α*_*i*_, and an increasing function of the phosphatase activity, *β*_*i*_. In this case, increasing the kinase activity downstream can shield upstream changes, and then lead to duration robustness against changes in the phosphatase activity in upstream modules. This is interesting as changes appear to be propagated upstream. This type of downstream-to-upstream perturbation transference was reported in the steady-state concentration of linear cascades as *retroactivity* [20], whereas our upstream propagation in the duration robustness is a different type of retroactivity since it is concerned with the initial condition for shielding upstream parameter changes.

### Biological relevance

Our results showed that within the range of a biologically relevant parameter set of a MAPK signaling pathway reported in [15], the duration and temporal profile of a strongly activated response are robust against perturbations in the phosphatase activities in the first and last modules. Past research has shown that temporal profiles of signaling cascades upon different inputs can lead to drastically different behaviors in cells. As mentioned earlier, transient versus sustained activation leads to different developmental responses [21, 22], and the behavior of transients in the p53 pathway is important to understanding certain types of cancer [23]. Our theory of dynamics robustness suggests that the transients involved in such decision processes can be robust to internal fluctuations in the concentrations of enzymes. We claim that stronger kinase activities are important for generating robust temporal profiles and that such a relationship will be verified experimentally.

Whereas our results show that the response duration and profile can be robust to some parameters, sensitivity to other external control parameters is necessary. Pathways need to be sensitive to certain parameters to function in the signaling process. Compatibility between robustness and plasticity (or sensitivity) to external changes is a basic characteristic of a biological system. In biological clocks, it is represented as reciprocity between robustness of period and plasticity in phase of oscillation [24], and it will be important to uncover a principle of how signaling pathways achieve both sensitivity to certain inputs while keeping robustness to other external changes.

The fast-slow-fast organization of the kinetics in the three-stage cascade adopted in the present model is not necessary for dynamics robustness since it is observed in two-stage cascades as well. We looked at other parameter setups in linear cascades and their results intuitively agreed with the results in this paper; the rate-limiting module tends to control the duration and the other modules display robustness under the constraints discussed. Whether the fast-slow-fast organization is a byproduct of another selected property or is selected for a beneficial trait regarding dynamics robustness is unknown. However, one possible benefit is the emergence of a plateauing response as observed in Figs 5(A) and 2(A). In this plateauing behavior, the response remains in a quasi-steady state before decaying exponentially. It is possible that a three-stage linear cascade may be used to store information in one of these reliably timed plateaus. Dynamics robustness may explain the reliability of the response, but future work is needed to explain the mechanism of the plateauing response and its relationship with dynamics robustness. This type of plateauing response has been discussed before as kinetic memory in other biochemical systems [25, 26] and such memory is also expected in a linear cascade with a fast-slow-fast organization.

As a design principle, a signaling cascade with the conditions discussed previously are ideal for robust transients and this parameter organization is reflected in [15]. Since reliably timed transients are useful in signal processing, robustness would make such properties evolutionarily feasible. Indeed, a repetitive cascade structure would be easily evolvable by gene duplication [27, 28], wherein the function of the original cascade is safeguarded by robust parameters.

The present paper focused on linear signaling cascades because of the recent interest in their temporal dynamics, but our idea of dynamics robustness can be generalized to any biochemical network. Two common properties of the cascade architectures we examined were mass conservation and an active molecule working as a kinase downstream. This suggests that such robustness can be achieved by similar designs such as the two-component signaling network in bacteria, and may be a universal feature in biological signaling cascades via protein modifications. There are some biochemical processes which are known to be reliably timed, such as lysis of bacteria and chromosome segregation during mitosis [29, 30], and these reliably-timed processes might be considered as a demonstration of dynamics robustness. Such robustness should be an essential property to many biological systems, and the expansion of the present formulation will provide a future fruitful area of research.

The concept and explicit results of *dynamics robustness* we have presented here should be timely and of importance. In many biological phenomena, the time course, such as the response against external stimuli or the developmental process, is crucially important, and must be sufficiently robust to perturbations. This point has been noted before, but so far there is no theory for such dynamics robustness. For example, the scale invariance of time course has gathered much attention as fold-change detection [31]. Dynamics robustness is concerned with the insensitivity to external changes rather than the scale invariance of time courses, and does not require strong constraints as imposed in the fold-change detection. Dynamics robustness can appear in a cascading system in general by shielding upstream parameter changes not only restricted to linear cascades, but systems with crosstalk as well. Thus it will have broader impacts and applications.

Future work will be done to incorporate more complicated design elements, such as the addition of feedback loops. It will be important to examine other cascading networks such as transcription regulatory cascades. For example, robustness of sensory response is discussed in transcription networks where feedback loop network motfis work as a rate limiting process [32]. It will be important to incorporate the feedback motifs with the present shielding mechanism in the cascade to design a network for dynamics robustness applicable to transcription and other pathways.

We demonstrated dynamics robustness in standard models of signal transduction. As these models are based on experimental data, and agree rather well with them, our dynamics robustness can be straightforwardly confirmed in cell-signaling experiments. Also considering the generality of our results, many other experimental topics will benefit from dynamics robustness.

## Models and methods

We looked at different models of varying complexity. Although we use the nomenclature of kinases and phosphatases to represent the activating enzymes and deactivating enzymes, our model can be applied generally to any linear signaling cascade. We used mass action kinetics to simulate the chemical reactions, and all of our equations were solved using MATLAB’s (version R2009a) built-in numerical integrator ode15s.

### Heinrich model

In the Heinrich model described in [16] and diagrammed in Fig 1, the receptor, *E*_0_, converts *M*_0_ to $M_{0}^{p}$, and $M_{0}^{p}$ converts *M*_1_ to $M_{1}^{p}$, and $M_{1}^{p}$ converts *M*_2_ to $M_{2}^{p}$, which is the output. The second order reaction rate at which *M*_*i*_ is activated is ${\bar{\alpha }}_{i}$, and the first order deactivation rate for $M_{i}^{p}$ is *β*_*i*_. We assume that after an initial, constant stimulus and equilibration of the system, the receptor is immediately shut off and the system relaxes.

There are a few major simplifying assumptions in this model that make it useful for examining the qualitative behavior of linear cascades. The assumptions are that the intermediate complexes formed by each kinase-substrate pair is negligible, that the backward reaction from the complexes is insignificant, and that the active phosphatase concentration is nearly constant. This means that the phosphatases and the intermediate complexes can be ignored, the desphosphorylation rate can be expressed as a first-order reaction rate, and that the sum of the inactive and active forms of each substrate is constant, i.e., $M_{i}+M_{i}^{p}=M_{i}^{tot}$ where $M_{i}^{tot}$ represents the total amount of substrate *M*_*i*_. Although these assumptions ignore some details, they enable us to analyze the models mathematically while still capturing the overall behavior of a signaling cascade. The corresponding set of equations post-stimulus is:

$$\begin{array}{cc} {\dot{M}}_{0}^{p} & =-\beta_{0}M_{0}^{p},\\ {\dot{M}}_{1}^{p} & ={\bar{\alpha }}_{1}M_{0}^{p}\left(M_{1}^{tot}-M_{1}^{p}\right)-\beta_{1}M_{1}^{p},\\ {\dot{M}}_{2}^{p} & ={\bar{\alpha }}_{2}M_{1}^{p}\left(M_{2}^{tot}-M_{2}^{p}\right)-\beta_{2}M_{2}^{p},\\ M_{0}^{p}\left(0\right) & ={\tilde{M}}_{0}=\frac{{\bar{\alpha }}_{0}E_{0}^{init}M_{0}^{tot}}{{\bar{\alpha }}_{0}E_{0}^{init}+\beta_{0}},\\ M_{i}^{p}\left(0\right) & ={\tilde{M}}_{i}=\frac{{\bar{\alpha }}_{i}{\tilde{M}}_{i-1}M_{i}^{tot}}{{\bar{\alpha }}_{i}{\tilde{M}}_{i-1}+\beta_{i}}. \end{array}$$ (6)

We note that Eq 6 is equivalent to Heinrich’s model, albeit with a slightly different form. An equivalent normalized model, i.e., where $m_{i}^{p}\left(0\right)=1$, has the form:

$$\begin{array}{cc} {\dot{m}}_{0}^{p} & =-\beta_{0}m_{0}^{p},\\ {\dot{m}}_{1}^{p} & =\alpha_{1}g_{0}m_{0}^{p}\left(g_{1}^{-1}-m_{1}^{p}\right)-\beta_{1}m_{1}^{p},\\ {\dot{m}}_{2}^{p} & =\alpha_{2}g_{1}m_{1}^{p}\left(g_{2}^{-1}-m_{2}^{p}\right)-\beta_{2}m_{2}^{p}, \end{array}$$ (7)

where $\alpha_{0}={\bar{\alpha }}_{0}E_{0}^{init}$ and $\alpha_{i}={\bar{\alpha }}_{i}M_{i-1}^{tot}$ are the effective kinase activities.

### Logarithmic gain

How robust a system is to a perturbation in a parameter is quantitatively measured by logarithmic gain. If one plots the dependent variable (say *y*) against a parameter (say *x*) on a log-log scale, then the logarithmic gain at a point is the slope of the tangent at that point. In other words, the logarithmic gain at a point *x*_0_ is $\frac{\partial \log(y)}{\partial \log(x)}$ at *x*_0_. If *y* is inversely proportional to *x*, then the logarithmic gain will be −1. This concept has been used in systems biology to measure the robustness of steady-state concentration levels and transition times [33, 34], but here we use it to measure how robust the half-life of a linear signaling cascade is against parameter changes, which, to the best of our knowledge has not been done before.

## Supporting information

S1 Appendix Additional Information. We provide tables of the parameters used in our simulations and equation derivations.
(PDF)
