# Supplementary material for: Dynamics robustness of cascading systems
Source: PLoS Comput Biol. 2017 Mar 13;13(3):e1005434. doi: 10.1371/journal.pcbi.1005434 (PMC5367838; doi:10.1371/journal.pcbi.1005434)
Supplement: S1 Appendix — We provide tables of the parameters used in our simulations and equation derivations. (PDF) [file pcbi.1005434.s001.pdf]

Supporting Information

1 Huang and Ferrell Model

In the original Huang and Ferrell (HF) model, an input stimuli, E_0, activates a MAP-kinase at the top layer. We labeled this substrate, M_0. We note that our labels are different from the labels Huang and Ferrell used so we could be consistent with other models in this paper. The activated form, M_0^p, can be deactivated by P_0, or it can go on to activate a MAP-kinase downstream, M_1. Using *Xenopus* oocyte extracts as a model, Huang and Ferrell assumed that two phosphorylation events are needed to activate M_1 and the next MAP-kinase downstream, M_2. Likewise, the activated form, M_1^{pp}, is deactivated by the phosphatase P_1 in a two step process, and M_2^{pp} is dephosphorylated by P_2 in a two step process. The M_2^{pp} concentration is regarded as the response. The rate equations are derived by mass action assumptions. The kinetic parameters that Huang and Ferrell used, which reflect actual parameters experimentally derived, are listed in Table A. To best illustrate the concept of dynamics robustness, the basal phosphatase concentrations we used are slightly different from the original Huang and Ferrell parameters.

As in the Heinrich model, we gave the system an initial input with strength E_0^{init}. We allowed the system to reach an equilibrium state, and then removed the stimulus at a rate of λ. Because the HF model assumes complex formation, it was necessary to specifically remove the stimulus instead of simply setting it equal to zero as was done in the Heinrich model.

The stoichiometry of the Huang-Ferrell model [1] with enzyme destruction is given by:

$$
\begin{aligned}
E_0 &\xrightarrow{\lambda} \varnothing, \\
E_0 + M_0 &\underset{d_0}{\overset{a_0}{\rightleftarrows}} C_0 \xrightarrow{k_0} E_0 + M_0^p, \\
P_0 + M_0^p &\underset{\tilde{d}_0}{\overset{\tilde{a}_0}{\rightleftarrows}} X_0 \xrightarrow{\tilde{k}_0} P_0 + M_0, \\
M_0^p + M_1 &\underset{d_1}{\overset{a_1}{\rightleftarrows}} C_1 \xrightarrow{k_0} M_0^p + M_1^p, \\
P_1 + M_1^p &\underset{\tilde{d}_1}{\overset{\tilde{a}_1}{\rightleftarrows}} X_1 \xrightarrow{\tilde{k}_1} P_1 + M_1,
\end{aligned}
$$

$$M_0^p + M_1^p \underset{d_2}{\overset{a_2}{\rightleftarrows}} C_2 \xrightarrow{k_2} M_0^p + M_1^{pp},$$

$$P_1 + M_1^{pp} \underset{\tilde{d}_2}{\overset{\tilde{a}_2}{\rightleftarrows}} X_2 \xrightarrow{\tilde{k}_2} P_1 + M_1^p,$$

$$M_1^{pp} + M_2 \underset{d_3}{\overset{a_3}{\rightleftarrows}} C_3 \xrightarrow{k_3} M_1^{pp} + M_2^p,$$

$$P_2 + M_2^p \underset{\tilde{d}_3}{\overset{\tilde{a}_3}{\rightleftarrows}} X_3 \xrightarrow{\tilde{k}_3} P_2 + M_2,$$

$$M_1^{pp} + M_2^p \underset{d_4}{\overset{a_4}{\rightleftarrows}} C_4 \xrightarrow{k_4} M_1^{pp} + M_2^{pp},$$

$$P_2 + M_2^{pp} \underset{\tilde{d}_4}{\overset{\tilde{a}_4}{\rightleftarrows}} X_4 \xrightarrow{\tilde{k}_4} P_2 + M_2^p.$$

and the corresponding mass-action system is given by:

$$\dot{E}_0 = -\lambda E_0 - a_0 E_0 M_0 + (d_0 + k_0) C_0,$$

$$\dot{M}_0 = -a_0 E_0 M_0 + d_0 C_0 + \tilde{k}_0 X_0,$$

$$\dot{C}_0 = a_0 E_0 M_0 - (d_0 + k_0) C_0,$$

$$\dot{M}_0^p = k_0 C_0 - \tilde{a}_0 P_0 M_0^p + \tilde{d}_0 X_0 - a_1 M_0^p M_1 + (d_1 + k_1) C_1 - a_2 M_0^p M_1^p + (d_2 + k_2) C_2,$$

$$\dot{P}_0 = -\tilde{a}_0 P_0 M_0^p + (\tilde{d}_0 + \tilde{k}_0) X_0,$$

$$\dot{X}_0 = \tilde{a}_0 P_0 M_0^p - (\tilde{d}_0 + \tilde{k}_0) X_0,$$

$$\dot{M}_1 = -a_1 M_0^p M_1 + d_1 C_1 + \tilde{k}_1 X_1,$$

$$\dot{C}_1 = a_1 M_0^p M_1 - (d_1 + k_1) C_1,$$

$$\dot{M}_1^p = k_1 C_1 - \tilde{a}_1 P_1 M_1^p + \tilde{d}_1 X_1 + \tilde{k}_2 X_2 - a_2 M_0^p M_1^p + d_2 C_2,$$

$$\dot{C}_2 = a_2 M_0^p M_1^p - (d_2 + k_2) C_2,$$

$$\dot{M}_1^{pp} = k_2 C_2 - \tilde{a}_2 P_1 M_1^{pp} + \tilde{d}_2 X_2 - a_3 M_1^{pp} M_2 + (d_3 + k_3) C_3 - a_4 M_1^{pp} M_2^p + (d_4 + k_4) C_4,$$

$$\dot{P}_1 = -\tilde{a}_1 P_1 M_1^p + (\tilde{d}_1 + \tilde{k}_1) X_1 - \tilde{a}_2 P_1 M_1^{pp} + (\tilde{d}_2 + \tilde{k}_2) X_2,$$

$$\dot{X}_1 = \tilde{a}_1 P_1 M_1^p - (\tilde{d}_1 + \tilde{k}_1) X_1,$$

$$\dot{X}_2 = \tilde{a}_2 P_1 M_1^{pp} - (\tilde{d}_2 + \tilde{k}_2) X_2,$$

$$
\begin{aligned}
\dot{M}_2 &= -a_3 M_1^{pp} M_2 + d_3 C_3 + \tilde{k}_3 X_3, \\
\dot{C}_3 &= a_3 M_1^{pp} M_2 - (d_3 + k_3) C_3, \\
\dot{M}_2^p &= k_3 C_3 - \tilde{a}_3 P_2 M_2^p + \tilde{d}_3 X_3 + \tilde{k}_4 X_4 - a_4 M_1^{pp} M_2^p + d_4 C_4, \\
\dot{C}_4 &= a_4 M_1^{pp} M_2^p - (d_4 + k_4) C_4, \\
\dot{M}_2^{pp} &= k_4 C_4 - \tilde{a}_4 P_2 M_2^{pp} + \tilde{d}_4 X_4, \\
\dot{P}_2 &= -\tilde{a}_3 P_2 M_2^p + (\tilde{d}_3 + \tilde{k}_3) X_3 - \tilde{a}_4 P_2 M_2^{pp} + (\tilde{d}_4 + \tilde{k}_4) X_4, \\
\dot{X}_3 &= \tilde{a}_3 P_2 M_2^p - (\tilde{d}_3 + \tilde{k}_3) X_3, \\
\dot{X}_4 &= \tilde{a}_4 P_2 M_2^{pp} - (\tilde{d}_4 + \tilde{k}_4) X_4,
\end{aligned}
$$

$$
\begin{aligned}
M_0^{tot} &= M_0 + M_0^p + C_0 + C_1 + C_2 + X_0, \\
P_0^{tot} &= P_0 + X_0, \\
M_1^{tot} &= M_1 + M_1^p + M_1^{pp} + C_1 + C_2 + C_3 + C_4 + X_1 + X_2, \\
P_1^{tot} &= P_1 + X_1 + X_2, \\
M_2^{tot} &= M_2 + M_2^p + M_2^{pp} + C_3 + C_4 + X_3 + X_4, \\
P_2^{tot} &= P_2 + X_3 + X_4,
\end{aligned}
$$

where the last six equations represent the conserved quantities.

2 Heinrich and HF Model Parameters

Parameter	Units	Value From [1]	Base Value for Our Results
E_0^{init}	μM	$--$	3×10^{-3}
M_0^{tot}	μM	3×10^{-3}	3×10^{-3}
P_0^{tot}	μM	3×10^{-4}	5×10^{-3}
M_1^{tot}	μM	1.2	1.2
P_1^{tot}	μM	3×10^{-4}	3×10^{-4}
M_2^{tot}	μM	1.2	1.2
P_2^{tot}	μM	0.12	0.01
λ	$(\text{min})^{-1}$	$--$	0, 100
a_i, $\tilde{a}_i$	$(\mu M \cdot \text{min})^{-1}$	1000	1000
d_i, $\tilde{d}_i$	$(\text{min})^{-1}$	150	150
k_i, $\tilde{k}_i$	$(\text{min})^{-1}$	150	150

Table A: Parameters used for the Huang and Ferrell model. The original parameters from [1] are displayed in the third column. The parameters used for our results are within a reasonable range of the original parameter set, but are slightly different to better exemplify dynamics robustness.

Parameter	Value	Parameter	Value
E_0^{init}	1	M_0^{tot}	1
M_1^{tot}	1	M_2^{tot}	1
α_0	1	β_0	0.5
α_1	10^{-1}	β_1	10^{-2}
α_1	10^{2}	β_2	2

Table B: Parameters used for the Heinrich model [2] . These parameters were chosen to reflect the same organization of the kinetics from the Huang and Ferrell model.

3 Derivation of Equations

3.1 The largest value of β_i at which the system is activated

The equations for β_i^{max} can be easily derived from the g_i functions, and they also demonstrate how the kinase activities only alter the β_i^{max} values and the corresponding regions of duration robustness upstream.

$$\beta_0^{max} = \alpha_0 \left(\frac{\alpha_1}{\beta_1} \left(\frac{\alpha_2}{\beta_2} - 1 \right) - 1 \right),$$
$$\beta_1^{max} = \alpha_1 \left(\frac{\alpha_2}{\beta_2} - 1 \right) \left(\frac{1}{1 + \frac{\beta_0}{\alpha_0}} \right),$$
$$\beta_2^{max} = \alpha_2 \left(\frac{1}{1 + \frac{\beta_1}{\alpha_1} \left(1 + \frac{\beta_0}{\alpha_0} \right)} \right).$$

3.2 Logarithmic Gains

In the main text it was shown that

$$\frac{\partial \log(\vartheta)}{\partial \log(\beta_i)} = \frac{1}{\log(2) + \log(c_k)} \frac{\partial \log(c_k)}{\partial \log(\beta_i)},$$

where i and k are such that β_k is the minimum β value and $i \neq k$. Our goal is to determine under what conditions is $\left| \frac{\partial \log(\vartheta)}{\partial \log(\beta_i)} \right| \ll 1$.

Case I: β_0 is minimum β value, and duration robustness with respect to β_1. First, let us consider the case when β_0 is the minimum β value, and under what conditions will the duration be robust to changes in β_1. We have that

$$c_0 = \frac{\alpha_1 \alpha_2 g_0}{g_2 (\beta_2 - \beta_0)(\beta_1 - \beta_0)}.$$

Hence,

$$\frac{\partial \log(c_0)}{\partial \log(\beta_1)} = -\frac{\partial \log(\beta_1 - \beta_0)}{\partial \log(\beta_1)} - \frac{\partial \log(g_2)}{\partial \log(\beta_1)} = \frac{-\beta_1}{\beta_1 - \beta_0} - \frac{\partial \log(g_2)}{\partial \log(\beta_1)}.$$

Therefore,

$$\frac{\partial \log(\vartheta)}{\partial \log(\beta_1)} = \frac{-1}{\log(2)+\log(c_0)} \left(\frac{\beta_1}{\beta_1-\beta_0} + \frac{\partial \log(g_2)}{\partial \log(\beta_1)} \right).$$

Thus, to minimize $\left|\frac{\partial \log(\vartheta)}{\partial \log(\beta_1)}\right|$ when β_1 is sufficiently larger than β_0, it is necessary to maximize c_0 and to minimize $\left|\frac{\partial \log(g_2)}{\partial \log(\beta_1)}\right|$. Now,

$$\frac{\partial \log(g_2)}{\partial \log(\beta_1)} = \frac{-\left(1+\frac{\beta_0}{\alpha_0}\right)\beta_1\beta_2 g_2}{\alpha_1\alpha_2}$$

Hence, if

$$\beta_1\beta_2 \ll \alpha_1\alpha_2,$$

then $\left|\frac{\partial \log(\vartheta)}{\partial \log(\beta_1)}\right|$ will be minimized.

Case II: β_0 is minimum β value, and duration robustness with respect to β_2. A similar argument as above shows that the condition needed is

$$\beta_2 \ll \alpha_2.$$

Case III: β_1 is minimum β value, and duration robustness with respect to β_0. In this case, we have that

$$c_1 = \frac{\alpha_2}{g_2(\beta_2-\beta_1)} \left(g_1 + \frac{\alpha_1 g_0}{\beta_0-\beta_1} \right).$$

Hence,

$$\frac{\partial \log(c_1)}{\partial \log(\beta_0)} = -\frac{\partial \log(g_2)}{\partial \log(\beta_0)} + \frac{\partial}{\partial \log(\beta_0)} \left(g_1 + \frac{\alpha_1 g_0}{\beta_0-\beta_1} \right).$$

It can be shown that the second term is equal to

$$-\left[\frac{\beta_0\beta_1}{\alpha_0\alpha_1}\right] \left(\frac{g_1}{1+\frac{\alpha_1 g_0}{(\beta_0-\beta_1)g_1}} \right) - \left[\frac{\beta_0}{\beta_0-\beta_1} - \frac{\beta_0}{\beta_0+\alpha_0}\right] \left(\frac{1}{1+\frac{g_1(\beta_0-\beta_1)}{\alpha_1 g_0}} \right).$$

The terms in the parentheses are all less than 1. Hence, $|\log(2)+\log(c_1)|$ can be maximized and $\frac{\partial \log(c_k)}{\partial \log(\beta_i)}$ can be minimized if

$$\beta_0\beta_1\beta_2 \ll \alpha_0\alpha_1\alpha_2.$$

Case IV: β_1 is minimum β value, and duration robustness with respect to β_2. We have that

$$\frac{\partial \log(c_1)}{\partial \log(\beta_2)} = -\frac{\partial \log(\beta_2 - \beta_1)}{\partial \log(\beta_2)} - \frac{\partial \log(g_2)}{\partial \log(\beta_2)},$$

and the same argument as Case I can be used to show the condition needed is

$$\beta_2 \ll \alpha_2.$$

Case V and VI: β_2 is minimum β value, and duration robustness with respect to β_0, β_1. We have that

$$c_2 = 1 + \frac{\alpha_2}{g_2(\beta_1 - \beta_2)} \left(g_1 + \frac{\alpha_1 g_0}{\beta_0 - \beta_2} \right).$$

A similar analysis as above shows that the condition needed is that

$$\beta_i \beta_{i+1} \cdots \beta_N \ll \alpha_i \alpha_{i+1} \cdots \alpha_N.$$

Summary In all, given the constraint that the cascade is considered activated, the constraint on the initial conditions can be grouped together as:

$$\frac{\beta_N}{\alpha_N} \left(1 + \frac{\beta_{N-1}}{\alpha_{N-1}} \left(\cdots \left(1 + \frac{\beta_0}{\alpha_0} \right) \right) \right) < 1,$$

$$\exists k \geq i \text{ such that } \beta_k \ll \alpha_k.$$

References

[1] Huang CY, Ferrell JE. Ultrasensitivity in the mitogen-activated protein kinase cascade. Proc Natl Acad Sci U S A. 1996;93(19):10078–10083.

[2] Heinrich R, Neel BG, Rapoport TA. Mathematical models of protein kinase signal transduction. Mol Cell. 2002;9(5):957 – 970.
